# Systematic screen for mutants resistant to TORC1 inhibition in fission yeast reveals genes involved in cellular ageing and growth

**DOI:** 10.1242/bio.20147245

**Published:** 2014-01-17

**Authors:** Charalampos Rallis, Luis López-Maury, Teodora Georgescu, Vera Pancaldi, Jürg Bähler

**Affiliations:** 1 Department of Genetics, Evolution and Environment, and Institute of Healthy Ageing, Darwin Building, University College London, Gower Street, London WC1E 6BT, UK; 2 Spanish National Cancer Research Centre (CNIO), 28029 Madrid, Spain; * Present address: Instituto de Bioquímica Vegetal y Fotosíntesis, Universidad de Sevilla-C.S.I.C., 41092 Sevilla, Spain

**Keywords:** TOR, *S. pombe*, Cell growth, Chronological lifespan, Oxidative stress, S6 kinase Sck2, Protein translation

## Abstract

Target of rapamycin complex 1 (TORC1), which controls growth in response to nutrients, promotes ageing in multiple organisms. The fission yeast *Schizosaccharomyces pombe* emerges as a valuable genetic model system to study TORC1 function and cellular ageing. Here we exploited the combinatorial action of rapamycin and caffeine, which inhibit fission yeast growth in a TORC1-dependent manner. We screened a deletion library, comprising ∼84% of all non-essential fission yeast genes, for drug-resistant mutants. This screen identified 33 genes encoding functions such as transcription, kinases, mitochondrial respiration, biosynthesis, intra-cellular trafficking, and stress response. Among the corresponding mutants, 5 showed shortened and 21 showed increased maximal chronological lifespans; 15 of the latter mutants showed no further lifespan increase with rapamycin and might thus represent key targets downstream of TORC1. We pursued the long-lived *sck2* mutant with additional functional analyses, revealing that the Sck2p kinase functions within the TORC1 network and is required for normal cell growth, global protein translation, and ribosomal S6 protein phosphorylation in a nutrient-dependent manner. Notably, slow cell growth was associated with all long-lived mutants while oxidative-stress resistance was not.

## Introduction

Lifespan is not invariably fixed but determined by genetic and environmental factors that are remarkably conserved across organisms. Budding yeast (*Saccharomyces cerevisiae*) has a long track record in providing fundamental insight into molecular mechanisms of cellular ageing (Kaeberlein, 2010). The distantly related fission yeast (*Schizosaccharomyces pombe*) is only just emerging as a complementary model for ageing; for example, recent studies have explored effects of glucose starvation on chronological lifespan (CLS), which leads to G2-arrest and death within a few days, while caloric restriction extends lifespan (Roux et al., 2009).

The conserved nutrient-responsive target of rapamycin (TOR) pathway regulates growth, metabolism and lifespan (Laplante and Sabatini, 2012; Wullschleger et al., 2006). TOR proteins are serine/threonine kinases found in two complexes: TORC1 and TORC2 (Wullschleger et al., 2006). TORC1 signaling promotes protein synthesis and ageing, and regulates growth in response to nitrogen or amino acid availability (Matsuo et al., 2007; Weisman, 2010). TORC1 promotes protein translation by phosphorylation of ribosomal S6 kinases and the eIF4E-binding protein (Laplante and Sabatini, 2012).

TORC1 can be inhibited by rapamycin, which forms an intracellular complex with the isomerase FKBP12 that then binds to the TOR kinase (Wullschleger et al., 2006). Rapamycin shows a strong inhibitory effect on cell growth in budding yeast (Heitman et al., 1991; Koltin et al., 1991). In fission yeast, on the other hand, rapamycin needs to be combined with caffeine for a strong inhibition of cell growth and division (Ikai et al., 2011; Rallis et al., 2013; Weisman and Choder, 2001). In budding yeast, TORC1 is also inhibited by caffeine, which leads to extended lifespan (Wanke et al., 2008), and deletion of TOR pathway genes or treatment with rapamycin prolongs the CLS (Powers et al., 2006). We have recently analysed the effects of caffeine and rapamycin on multiple cellular processes in fission yeast (Rallis et al., 2013). These drugs lead to diverse phenotypes that depend on TORC1 inhibition such as prolonged CLS, inhibition of global translation, and reprogramming of global gene expression mimicking nitrogen starvation.

TORC1 signaling has been intensely studied, but many aspects of this complex regulatory network remain elusive. Further insight into TOR function will be a driving force for the understanding of TORC1 control and cellular ageing, and for the design of inhibitors that target specific functions of the network. Genetic interactions such as synthetic lethality can identify proteins that impinge on a common essential function (Baryshnikova et al., 2010; Dixon et al., 2009). Similarly, drug-resistant mutants often show functional relationships with the cellular process targeted by the drug (Baryshnikova et al., 2010).

To uncover proteins related to TORC1 signaling and cellular ageing, we exploit here the growth inhibition by rapamycin and caffeine (Rallis et al., 2013; Takahara and Maeda, 2012) to screen a *S. pombe* deletion library for mutants resistant to the two drugs. Most of the identified mutants have not been implicated in TORC1 signaling before, and they often exhibit altered lifespans and growth rates. We perform additional functional analyses for one of the identified mutants that lacks the putative S6 kinase Sck2p. This study provides groundwork to guide future research in fission yeast and more complex organisms.

## Results

### Screen for deletion mutants resistant to rapamycin and caffeine

**Fig. 1.**
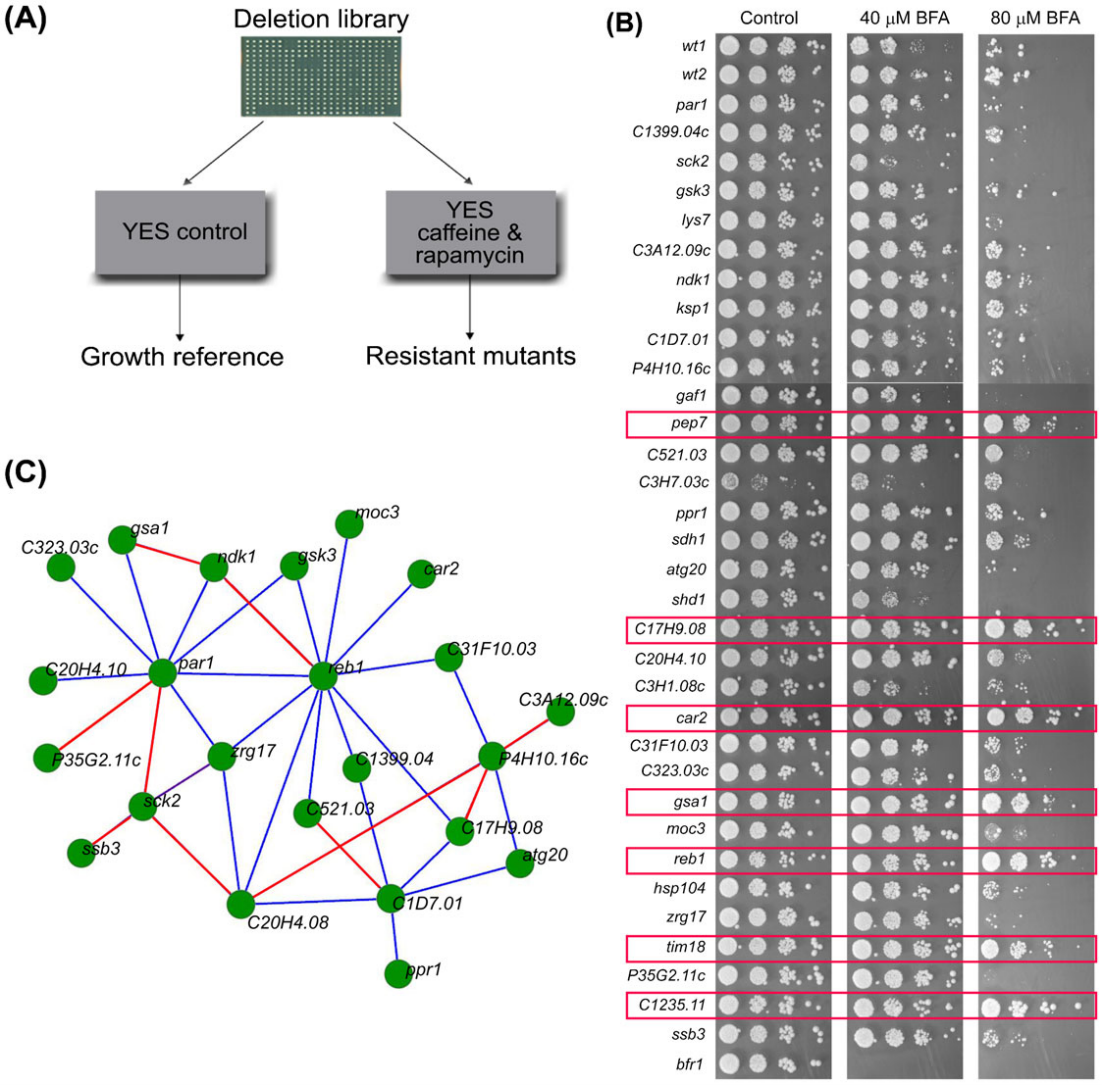
Screening strategy and lifespan of mutant cells. (A) Scheme for the genetic screen for deletion mutants resistant to the growth inhibition by rapamycin and caffeine. YES: yeast extract medium control. (B) Brefeldin A (BFA) sensitivity test using spotting assay for the 33 mutant strains identified in screen, using 2 BFA concentrations as indicated. Red outlines: mutants showing increased resistance to BFA. (C) Analysis of available genetic interactions (Ryan et al., 2012) for 23 of the mutants identified in screen. Blue and red lines represent negative and positive interactions, respectively.

Combined treatment of rapamycin and caffeine leads to a complete growth inhibition in fission yeast cells (Rallis et al., 2013; Takahara and Maeda, 2012). Using a library of 3005 deletion mutants, we screened ∼84% of all *S. pombe* non-essential genes to identify mutants resistant to the growth inhibition by the two drugs. As drug-resistant mutants often function within the drug-target pathway, we anticipated that this screen should uncover proteins involved in TORC1 signaling and cellular ageing. A series of different concentrations of rapamycin and caffeine was used in pilot experiments to determine the appropriate doses to inhibit the growth of most deletion strains (see also Rallis et al., 2013). A combination of 100 ng/ml rapamycin and 10 mM caffeine proved effective for screening. The library was spotted on control plates as a growth reference and on plates including rapamycin and caffeine (Fig. 1A). We conducted four independent biological repeats of the screen, and all mutants scored for drug resistance are shown in supplementary material Table S1. We focussed on 33 mutant hits that scored positive in at least 3 of the 4 repeats (supplementary material Table S2). We also assessed the library for sensitivity to caffeine (supplementary material Table S3); these results are not further discussed but are provided here as a resource for interested colleagues.

To verify the mutants identified from the deletion library, we independently confirmed all 33 mutant hits by PCR of the genomic-marker junctions created by the gene deletions. Moreover, we back-crossed all mutants to a wild-type strain to show co-segregation of the phenotype with the deletion marker, and three selected deletion mutants were independently recreated to check for consistency in phenotypes (Fig. 4A, Fig. 6A,B; Materials and Methods).

**Fig. 2.**
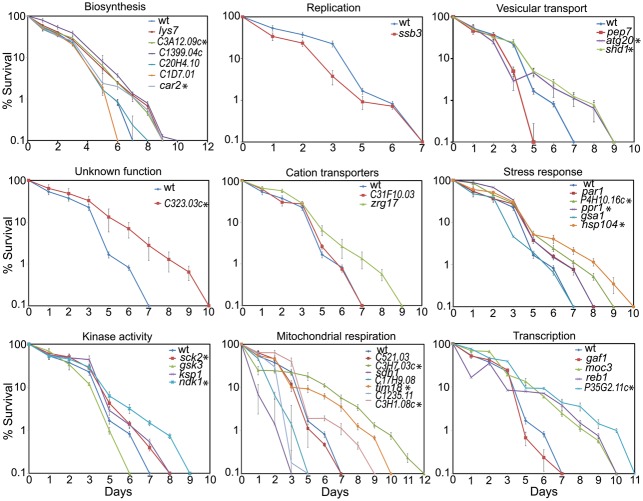
CLS assays for the 33 mutant strains identified in screen compared to wild-type (wt) strain. Time zero was set when cells reached stationary phase. Asterisks indicate long-lived mutants that showed no additional CLS extension with rapamycin treatment. These graphs highlight differences in maximal lifespan.

**Fig. 3.**
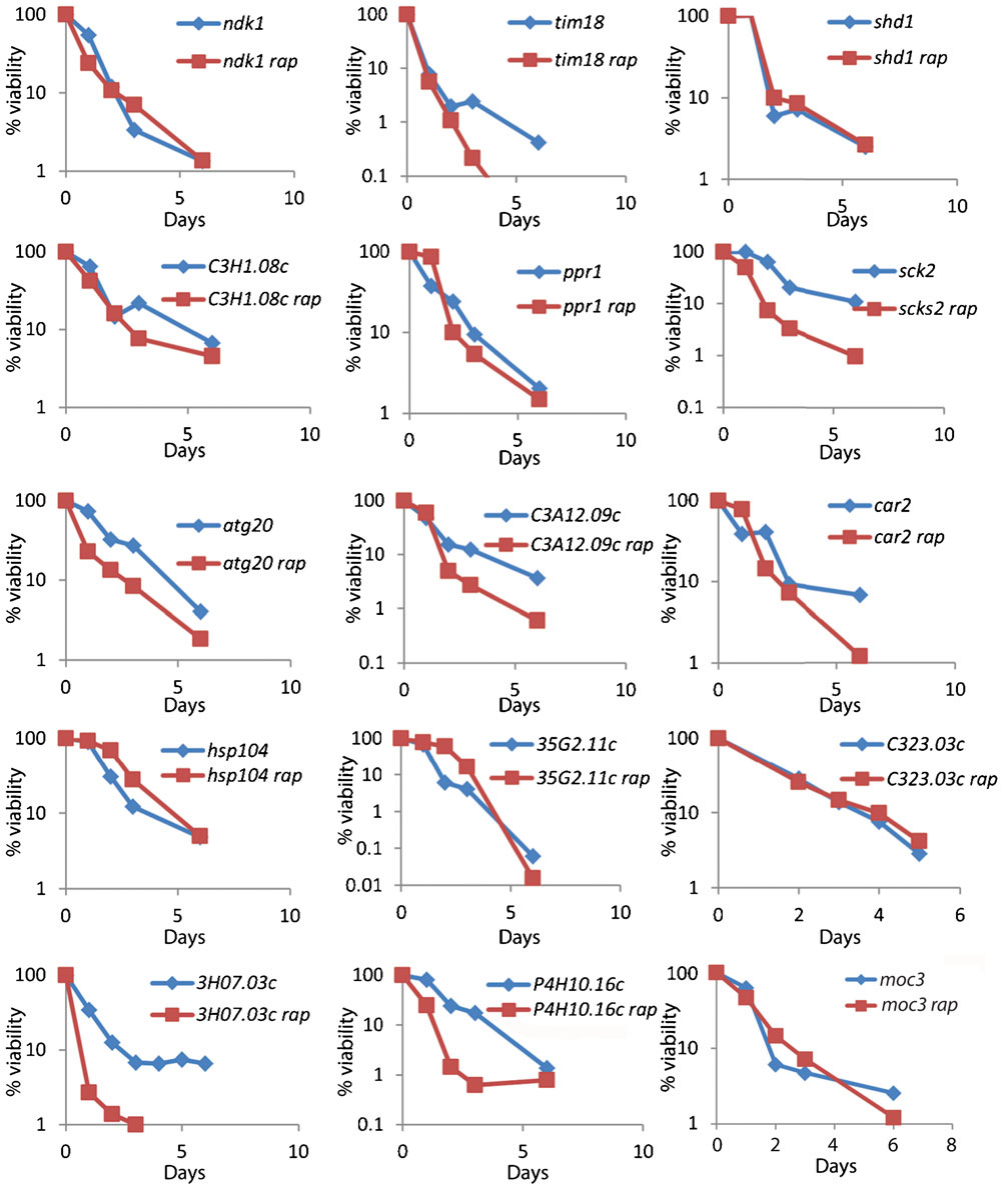
Comparison of CLS for long-lived mutants with (red) or without (blue) rapamycin treatment during growth phase. Only the 15 long-lived mutants that exhibited no additional extension of maximal CLS after rapamycin treatment are shown (p>0.05). Supplementary material Fig. S1 shows remaining 6 long-lived mutants that exhibited additional extension of maximal CLS when treated with rapamycin.

**Fig. 4.**
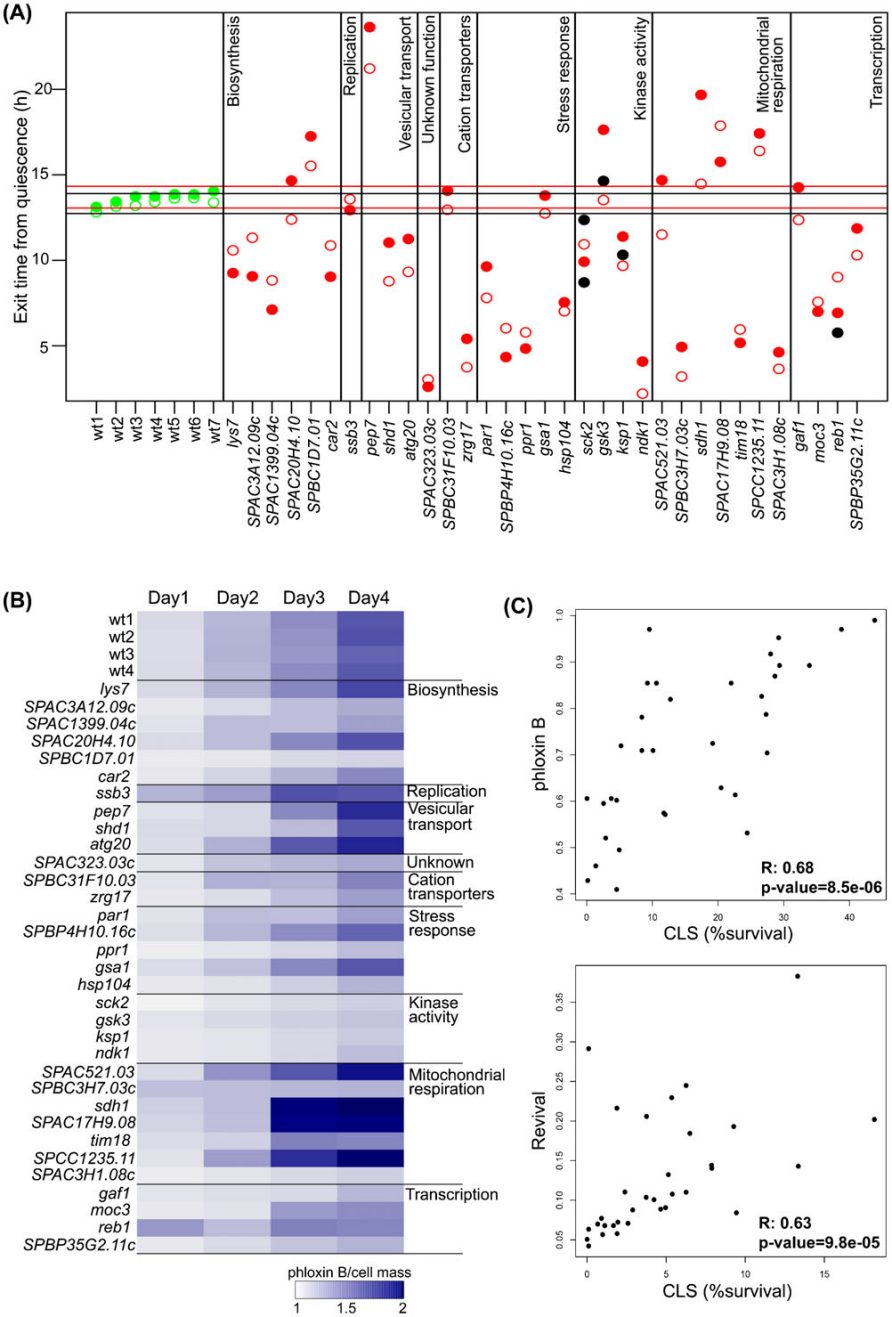
High-throughput assays to determine CLS. (A) Mutants examined for growth rate (using lag phase of non-stressed cells) and the time required to resume growth following 3 days in stationary phase. The lag phase in the absence of stress was subtracted from the lag phase observed following stationary phase. Mutant cultures taking shorter or longer times to exit quiescence contain fewer or more dead cells, indicating long- and short-lived mutants, respectively. Seven independent repeats each for 2 control strains were tested: *972 h^−^* (open green circles) and *ED668* (solid green circles). Black and red horizontal lines show 2 standard deviations from mean revival values for *972 h^−^* and *ED668*, respectively. Data shown are for 33 original deletion mutants from library as indicated (solid red circles), for corresponding mutants after back-crossing to wild type (open red circles), and for deletion mutants that we independently reconstructed (solid black circles). The average of 3 independent biological repeats is shown for each mutant data point. (B) Heat map showing phloxin B signals at 550 nm normalized with total cell mass (OD_600_) used to measure the signal at sequential days of stationary phase as indicated on top. Mutant cultures showing lower or higher phloxin B signals contain fewer or more dead cells, indicating long- and short-lived mutants, respectively. (C) Pairwise comparisons showing relationship between CLS (% survival) and cell-density normalized phloxin B signal (inverse ratio of phloxin B/OD_600_; top graph), as well as between CLS and inverse lag time (time to resume growth following 3 days in stationary phase; bottom graph). Pearson correlations together with p-values are indicated.

**Fig. 5.**
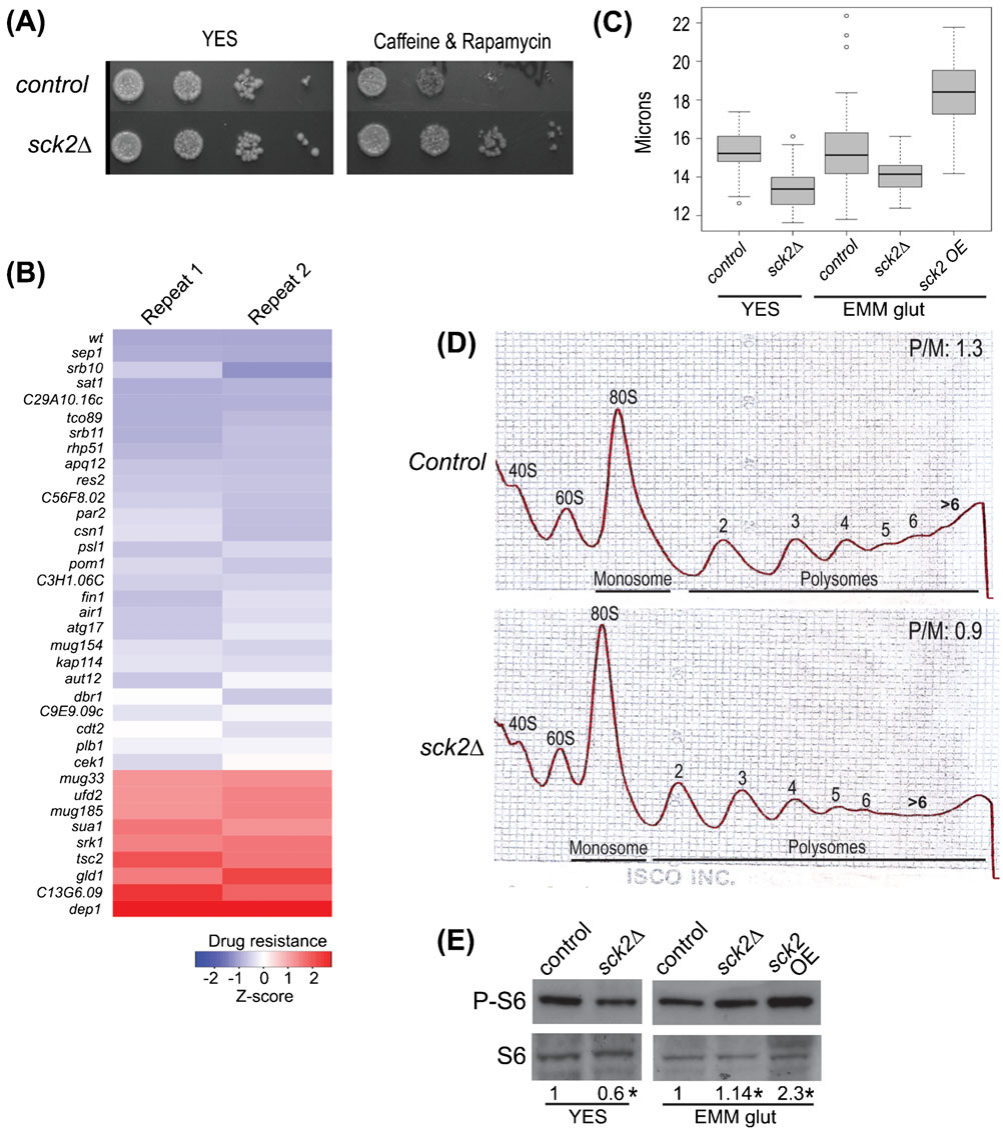
Sck2p functions in cell growth and protein translation. (A) Serial dilutions of wild-type and *sck2Δ* cells spotted on agar plates without (YES) or with drug treatment as indicated. (B) Heatmap of double mutants showing at least 1.5-fold lower (blue) or higher (red) caffeine and rapamycin resistance relative to *sck2Δ* single mutant. Data for 2 biological repeats are shown, with genes deleted along with *sck2Δ* indicated at left. Drug sensitivity/resistance was determined by colony size of double mutants relative to colony size of *sck2Δ* single mutant in presence of caffeine and rapamycin. Accordingly, the *sck2Δ* single mutant would be white in the color key used. (C) Cell size at division for wild type (control), *sck2Δ*, and cells overexpressing *sck2* (OE) in different media as indicated. *sck2Δ* cells are significantly smaller than wild-type cells in both YES and EMM (Wilcoxon, p∼2.2×10^−16^ and ∼1.1×10^−8^, respectively), while cells overexpressing *sck2* are significantly larger (Wilcoxon, p∼2.2×10^−16^). (D) Polysome profiles of wild-type and *sck2Δ* cells as indicated, grown in YES medium. A typical experiment is shown, with all data and significance provided in supplementary material Table S7. (E) Western showing phosphorylated S6 protein levels in wild type (control), *sck2Δ*, and cells overexpressing *sck2* (OE) grown in different media as indicated. Ratios of phosphorylated S6 levels in different strains relative to control have been calculated using ImageJ and are shown at bottom. Total S6 protein is used as loading control. Asterisks indicate statistical significance (p<0.05, paired t-tests using three biological repeats).

**Fig. 6.**
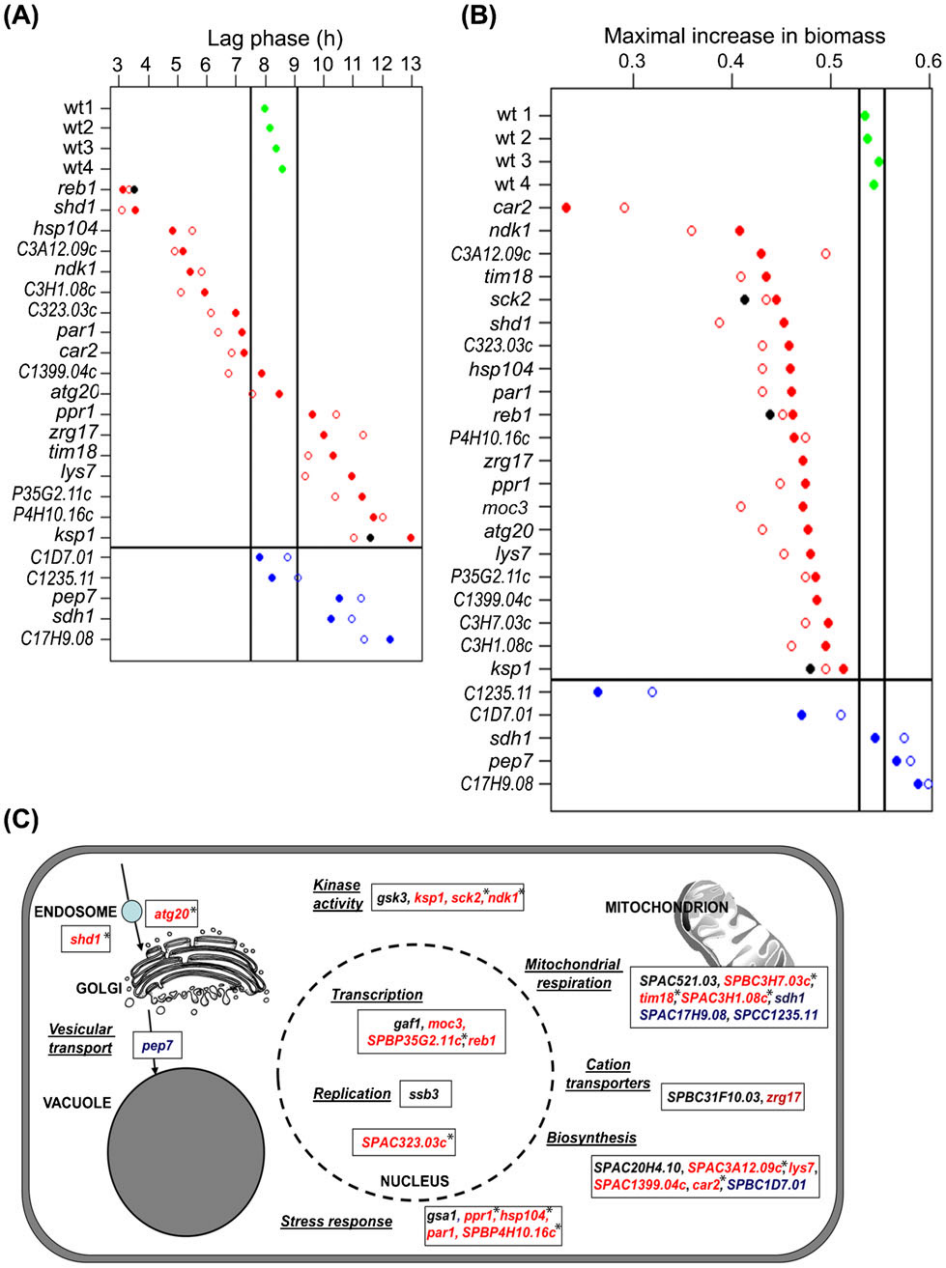
Stress resistance and growth phenotypes of mutants and gene functions identified in screen. (A) Lag phases after oxidative stress for mutants compared to wild type. Four independent repeats for wild type were measured (wt1–wt4, green circles), with vertical lines representing 2 standard deviations from mean. Data shown are for 18 original long-lived mutants from library as indicated (solid red circles), for corresponding mutants after back-crossing to wild type (open red circles), for deletion mutants that we independently reconstructed (solid black circles), for 5 original short-lived mutants (solid blue circles), and for corresponding back-crossed strains (open blue circles). The horizontal line separates long- from short-lived mutants. The average of 3 independent biological repeats is shown for each mutant data point. (B) Maximal increase in biomass (relative biomass change per min) of 21 long- and 5 short-lived deletion mutants as indicated, compared to the wild-type cells (colors and symbols as in Fig. 6A). Vertical black lines indicate 2 standard deviations from mean of 4 wild-type repeats. The average of 3 independent biological repeats is shown for each mutant data point. (C) Overview of functional categories identified in mutant screen, set in a cellular and functional context. Genes are color-coded for long- (red) and short-lived (blue) mutants, or mutants without change in CLS (black) compared to wild-type control. Asterisks indicate the long-lived mutants that show no additional CLS extension with rapamycin treatment (Fig. 3), which might represent key targets downstream of TORC1.

The 33 drug-resistant mutants were deleted for genes implicated in biosynthesis, mitochondrial functions, stress response, vacuolar trafficking and transcription. The hit list contained 5 genes (*par1*, *sck2*, *gsk3*, *ksp1*, *gaf1*) that have been linked to TORC1 signaling in other organisms (Georis et al., 2008; Inoki et al., 2006; Santhanam et al., 2004; Umekawa and Klionsky, 2012; Urban et al., 2007), suggesting that the screen uncovered relevant genes. As autophagy and meiotic differentiation are linked to TOR in *S. pombe* (Matsuo et al., 2007; Umekawa and Klionsky, 2012), two more hits could be rationalized with TOR function: *atg20*, encoding an autophagy-related protein, and *moc3*, encoding a meiotic transcription factor (Goldar et al., 2005). The latter shows a similar genetic-interaction signature as genes encoding TOR components (Ryan et al., 2012). There were similarities as well as notable differences to available data from *S. cerevisiae*. For example, budding yeast *ksp1* mutants are also resistant to TORC1 inhibition (Huber et al., 2009), whereas *whi2* mutants are sensitive unlike the orthologous *SPBP4H10.16c* mutants in fission yeast that were resistant.

Drug resistance could generally arise from overactive efflux pumps. The ABC transporter Bfr1p functions as the major caffeine exporter in fission yeast (Calvo et al., 2009). Overactive Bfr1p renders cells resistant to an inhibitor of intracellular transport, Brefeldin A (BFA), while cells lacking Bfr1p are hypersensitive to BFA (Nagao et al., 1995). We therefore examined whether our caffeine-resistant mutants are also BFA-resistant, revealing that 7 out of the 33 mutants were resistant to both drugs (Fig. 1B). Thus, 26 of our mutants could not be explained by altered caffeine efflux pump activity. Although the 7 BFA-resistant mutants seem less promising, some of them might still be related to TORC1 function as suggested by further analyses such as requirement for rapamycin-dependent lifespan extension (see below).

We examined the functional relationships among our mutant hits using a genetic interactome study for fission yeast (Ryan et al., 2012). For 25 out of the 33 hits, genetic interaction data were available. Notably, 23 out of these 25 genes showed at least one genetic interaction with other genes deleted in drug-resistant mutants (Fig. 1C). For example, *reb1* (one of the BFA-resistant mutants) showed 11 interactions and *par1* showed 9 genetic interactions with our other mutant hits. The occurrence of both positive and negative genetic interactions might reflect the complexity of the TORC1 signaling network that includes feedbacks, cross-talks, and partially redundant pathways. We calculated the number of genetic interactions amongst our 33 genes of interest that have been reported before and are listed in BioGRID (Chatr-Aryamontri et al., 2013). A conservative estimate suggests that the number of observed interactions is 6 times higher than expected by chance, indicating a significant enrichment of genetic interactions among our mutant hits (Permutation test; p = 0.0001). We conclude that the genes identified by the screen show substantial functional coherence.

### Mutants with altered lifespan

We measured the CLS for all 33 drug-resistant mutants identified in the screen compared to a control strain with same genetic background as the deletion library. Overall, 21 and 5 mutants showed increased and decreased maximal lifespans, respectively (Fig. 2; supplementary material Table S4), while 14 and 7 mutants showed increased and decreased medial lifespans, respectively (supplementary material Table S4). The proteins corresponding to the short- or long-lived mutants were not enriched for any particular functions. We also tested maximal lifespans of the long-lived mutants after rapamycin treatment (100 ng/ml): 15 out of the 21 mutants showed no additional extension of maximal CLS when treated with rapamycin (Fig. 3; supplementary material Table S5; indicated with asterisks in Fig. 2). The medial CLS for 4 of these mutants (*ppr1*, *hsp104*, *SPBP35G2.11c*, *car2*) was further increased following rapamycin treatment (p<0.05). The other 11 proteins may therefore be key targets for the full rapamycin-mediated effects on lifespan extension. The remaining 6 out of 21 long-lived mutants did exhibit an additional extension of maximal CLS when treated with rapamycin (supplementary material Fig. S1; p<0.05).

Besides determining CLS with classical colony forming assays, we also established two approaches more suitable for large-scale analyses. First, we defined the time (lag phase) taken for all mutants to resume rapid growth following dilution from OD_600_ 0.6 to 0.1. We then determined the time taken for the mutants to resume growth after 3 days in stationary phase (Powers et al., 2006). The lag phase observed in this experiment reflects a combination of growth rate and proportion of dead cells within the cultures. To obtain a measure of dead cells, we subtracted the lag phase obtained from the dilution experiment (which is based on live cells only) from the lag phase obtained in the stationary phase experiment. Owing to a lower proportion of live cells, short-lived mutant cultures are expected to resume growth later than wild-type cultures while long-lived cultures should resume growth earlier. Using this method, we validated all results for short- and long-lived mutants observed using the classical CLS assays (Fig. 4A, solid red circles). We further verified that the drug-resistant mutants from the Bioneer library contained deletions in the correct genes. To this end, we back-crossed all 33 mutants to a wild-type strain, which resulted in prototroph mutants showing co-segregation of the deletion marker with CLS phenotypes similar to those of the original library mutants (Fig. 4A, open red circles). Moreover, three mutants, *sck2*, *gsk3* and *reb1*, were independently recreated using standard PCR-based gene deletion (Bähler et al., 1998), with *sck2* being separately deleted using both *ura4* and *kanMX6* markers. The resulting four mutants also showed CLS phenotypes consistent with those of the corresponding library mutants (Fig. 4A, black circles), and in agreement with the classic CLS assays.

As a second approach, we measured the proportion of phloxin B-stained dead cells at successive days in stationary phase, normalised by the number of cells used to obtain the measurements (Fig. 4B). As expected, mutants that resumed growth more rapidly than wild type after three days in stationary phase showed lower phloxin B staining (Fig. 4A,B). The three approaches to determine lifespan correlated reasonably well with each other: mutants with long CLS tend to accumulate less phloxin B and resume growth earlier after stationary phase (Fig. 4C). However, there were a few discrepancies as might be expected; for example, not all cells that fail to grow become immediately phloxin B positive, and the time to resume growth will also depend on the growth kinetics of mutant cells. We propose that the time to resume growth and phloxin B staining provide effective alternative approaches to estimate lifespan for larger-scale assays, which can then be validated using the more time-consuming CLS assay.

Among the 26 short- and long-lived mutants (Fig. 2), only *sck2Δ* was previously reported to be long-lived in *S. pombe* (Chen and Runge, 2009; Roux et al., 2006); this mutant is deleted for the putative S6 kinase Sck2p and is further analysed below. Among the remaining 25 *S. pombe* mutants, only 2 orthologous mutants have been reported to show altered lifespans, and they are short-lived in budding yeast but long-lived in fission yeast (supplementary material Table S2). In addition, one of our 7 mutant hits that showed no effect on lifespan is long-lived in budding yeast (supplementary material Table S2). Thus, our screen uncovered mainly genetic factors not previously known to be involved in cellular ageing, highlighting the complementary insight provided by fission yeast.

### Sck2p kinase functions in cell growth and protein translation

We followed up the long-lived *sck2Δ* mutant with additional functional analyses. To verify the screen result, we first demonstrated that independently generated *sck2Δ* cells, unlike wild-type cells, showed no caffeine and rapamycin-induced growth inhibition (Fig. 5A).

To reveal global functional relationships of Sck2p, we performed a Synthetic Genetic Array analysis (SGA) (Baryshnikova et al., 2010). We crossed *sck2Δ* cells against all the other deletion mutants in the library. A control SGA using *ade6::natMX4* as a query (that does not alter the fitness of the deletion collection) was performed in parallel and used for colony normalisation. Genetic interactions in three independent experimental repeats were scored. This analysis revealed 112 and 79 genes that showed negative and positive genetic interactions, respectively (supplementary material Table S6). Negative genetic interactions were enriched for the following Gene Ontology categories (supplementary material Table S6): Transcription, RNA Metabolic Process, including genes for tRNA processing, and Signal Transducer Activity/Signal Transduction. The latter lists include genes of the Pka1p cAMP-dependent protein kinase signalling pathway. This finding is consistent with data showing that Sck2p affects lifespan independently of Pka1p (Chen and Runge, 2009; Roux et al., 2006). Positive genetic interactions were enriched for the Gene Ontology categories Organelle Organisation and Biogenesis, and Signal Transduction. The first category includes genes for chromatin organisation and chromosome segregation, while the second category includes genes for cell-cycle regulation, cell growth as well as core components of the TORC1 pathway (supplementary material Table S6). Furthermore, mutants in genes regulating autophagy (*aut12*, *atg17*) and phospholipid metabolism (*plb1*) also positively interacted with *sck2Δ*. As positive genetic interactions are often occurring within the same pathway, these data are fully consistent with Sck2p functioning within the TORC1 network.

To uncover genes required for the caffeine and rapamycin resistance of *sck2Δ* cells, we tested all double mutants that showed positive genetic interactions with *sck2Δ* for drug resistance. Interestingly, none of these positive interactors were drug resistant. This analysis uncovered 26 double mutants with similar drug sensitivity to wild-type cells (Fig. 5B). The corresponding genes are therefore required for the drug resistance of *sck2Δ* cells, including *tco89* encoding a core TORC1 component, autophagy-related genes, and several genes required in cell-cycle regulation. This analysis also uncovered 9 double mutants that actually showed higher drug resistance than *sck2Δ* single mutants (Fig. 5B). The corresponding genes include *tsc2*, encoding a negative regulator of TORC1 signalling, which could explain that double mutants can better overcome TORC1 inhibition by the drugs.

The genetic interaction data suggested that Sck2p is involved in cellular growth control and TORC1 function. Intriguingly, *sck2Δ* cells were smaller upon division, while overexpression of *sck2* resulted in increased cell size compared to wild type (Fig. 5C). These results indicate that Sck2p is involved in cell-size and/or cell-growth control, the latter being corroborated by a slow-growth phenotype of *sck2Δ* cells (Fig. 6B).

Although Sck2p is a putative S6 kinase based on sequence similarity, recent data indicate that it is not required for S6 ribosomal protein phosphorylation (Nakashima et al., 2012). To test whether Sck2p can control protein translation, we compared translational profiles of wild type with 3 independently generated *sck2Δ* strains (deleted with *ura4*, *kanMX6*, and *natMX6* markers), each experiment repeated 3 times independently (i.e. 3 wild-type and 9 *sck2Δ* repeats). The Polysome-to-Monosome (P/M) ratios in *sck2Δ* cells were consistently ∼30% lower than in wild-type cells (Fig. 5D; supplementary material Table S7), indicating fewer transcripts occupied with ribosomes in *sck2Δ* cells. This result indicates that Sck2p can positively affect global translation. This analysis was performed with rich YES media while Nakashima et al. did their experiments with minimal EMM media (Nakashima et al., 2012). We therefore analysed the phosphorylation status of S6 ribosomal proteins in different media. Interestingly, in YES, *sck2Δ* cells did show reduced S6 phosphorylation, while S6 phosphorylation was not affected in EMM glutamate (Fig. 5E). As reported (Nakashima et al., 2012), Sck2p was also not required for S6 phosphorylation in standard EMM (ammonium chloride as nitrogen source; data not shown). We conclude that Sck2p is involved in S6 phosphorylation in a medium-dependent manner, suggesting that it functions as an S6 kinase depending on available nutrients.

### Oxidative stress resistance is not linked with lifespan

Resistance to oxidative or other stresses has been associated with increased CLS (Roux et al., 2009; Zuin et al., 2010). We therefore determined the resistance to oxidative stress in the CLS mutant strains. Two results were notable (supplementary material Fig. S2). First, mutants deleted for functionally related genes did not always exhibit the same phenotypes; for example, of the two endosome genes, the *atg20Δ* mutant was sensitive to H_2_O_2_, while the *shd1Δ* mutant was resistant. Second, increased stress resistance was often not associated with increased CLS.

To quantitatively determine stress resistance, we assessed the growth dynamics of CLS mutants in the absence and presence of H_2_O_2_. Rapidly growing YES cultures were diluted to OD_600_ = 0.15 using YES. Cells were then grown in the absence or presence of the stressor. Oxidative stress led to an extended lag phase compared to untreated control cells before growth resumed (supplementary material Fig. S2). Analogous to Fig. 4A, stress-resistant or -sensitive mutants should therefore show shorter or longer lag phases before resuming growth, respectively, compared to wild-type cells. For long-lived mutants, we assessed the original deletion library strains (Fig. 6A, solid red circles), the prototroph back-crossed mutants (Fig. 6A, open red circles) as well as two deletion mutants that were independently reconstructed (Fig. 6A, black circles). For short-lived mutants, we assessed the original deletion library strains (Fig. 6A, solid blue circles) and the prototroph back-crossed mutants (Fig. 6A, open blue circles). As for CLS (Fig. 4A), the independent mutants deleted for the same genes showed similar phenotypes with respect to oxidative stress resistance. Notably, CLS and oxidative stress resistance were poorly correlated: long-lived mutants showed a broad and continuous phenotypic range, from more sensitive to more resistant to oxidative stress than wild type (Fig. 6A). The short-lived mutants seemed to show some tendency towards higher stress sensitivity, but our sample only included five such mutants. We conclude that the increased resistance to oxidative stress is not necessarily associated with longer CLS.

### Slow cell growth is linked to long lifespan

The CLS mutants reached different maximum cell densities, probably reflecting different efficiencies in utilising available nutrients (supplementary material Fig. S3). Relative to their CLS phenotypes, the mutants did not show any bias with respect to the maximal cell densities reached, which ranged from much lower to much higher than wild type (supplementary material Fig. S3). To further analyse the mutants' growth properties, we calculated the relative maximum biomass increase as an indicator for growth rate (Fig. 6B). Again, this analysis included the original deletion library strains (Fig. 6B, solid circles), prototroph back-crossed mutants (Fig. 6B, open circles), and three independently generated mutants (Fig. 6B, black circles). Notably, all long-lived mutants showed lower growth rates compared to wild type, while 3 of 5 short-lived mutants showed the same or higher growth rates. The 2 short-lived mutants with lower growth rates were deleted for the ortholog of the mitochondrial protein BRP44L (encoded by *SPCC1235.11*) and the prefoldin subunit 1 (encoded by *SPBC1D7.01*); these mutants may be severely impaired for energy production and protein quality control, respectively.

We conclude that in our mutants a slow growth rate was more directly linked to long CLS than oxidative stress resistance. Accordingly, the *S. pombe* mutants previously reported as long-lived are known to grow slowly, including *git3Δ*, *gpa2Δ*, *pka1Δ*and *sck2Δ* (Roux et al., 2009). The first three of these mutants are not thought to be directly involved in TORC1 function and were not identified in our screen. This result, together with the genetic interaction data (Fig. 1C), illustrates the specificity of the screen for TORC1-related factors.

## Discussion

A challenge of ageing research remains to uncover all genetic factors determining longevity. We exploited the TORC1-mediated growth inhibition of rapamycin and caffeine to uncover 33 deletion mutants that are resistant to the two drugs. Many of the corresponding genes are expected to function within the TORC1 network. Accordingly, most of the drug-resistant mutants showed altered CLS and growth characteristics. Fig. 6C provides an overview of the identified genes put in a cellular and functional context. Notably, our mutants showed abundant genetic interactions amongst themselves (Fig. 1C). This tight genetic interaction network suggests that most mutants identified in the screen are functionally related and coherent. Further studies will be required to determine any effects of calorie restriction on the identified mutants.

Follow-up analyses of the long-lived *sck2Δ* mutant revealed that Sck2p is important for cell size and growth, possibly via controlling global protein translation (Fig. 5). Sck2p is a putative member of S6 kinases, which play conserved roles in translational regulation and cellular ageing downstream of TORC1 (Laplante and Sabatini, 2012). Sck2p has been reported to not affect S6 ribosomal protein phosphorylation (Nakashima et al., 2012). We confirmed this finding for cells grown in minimal media with defined nitrogen source; however, when cells were grown in rich yeast extract medium containing complex nitrogen sources, Sck2p became important for normal levels of both polysomes and S6 phosphorylation (Fig. 5D,E). Together, these results suggest that Sck2p only contributes to global translational regulation under some nutritional conditions, like the availability of amino acids or other nitrogen sources. Notably, cells grew larger and showed increased S6 phosphorylation upon Sck2p overexpression, suggesting that Sck2p is sufficient to affect cellular growth and translational status. Unlike S6 phosphorylation, however, *sck2Δ* cells were smaller in both rich and minimal media (Fig. 5C), which could mean that Sck2p can affect cell size independently of translational control. Our genetic interaction data raise the possibility that Sck2p also relates to cell-size control and the coordination between cell growth and division. Interestingly, with glutamate as nitrogen source, Sck2p is required for Pmt1p-dependent methylation of tRNAs, which affects ribosome biogenesis and translation initiation (Becker et al., 2012). Sck2p itself becomes phosphorylated under nitrogen-rich conditions (Nakashima et al., 2010). Our results further suggest that Sck2p works within the TORC1 pathway: the *sck2Δ* mutant positively interacted with autophagy mutants and with *tco89Δ*, deleted for a core component of TORC1, while it negatively interacted with components of the Pka1p pathway. The latter data corroborate that Sck2p affects lifespan independently of cAMP/PKA1 pathway (Chen and Runge, 2009; Roux et al., 2006), and it suggests that Sck2p acts in parallel to, but outside of this pathway. Taken together, the data indicate that Sck2p functions within the TORC1 network by contributing to the control of global protein translation via different mechanisms in a nutrient-dependent manner.

Surprisingly, among our 25 other CLS mutants, only 2 are known CLS mutants in budding yeast, and they show opposite effects on lifespan in the two yeasts (supplementary material Table S2). This finding could reflect different biology of the distantly related yeasts such as the distinct cell-cycle arrest points: during glucose limitation budding yeast cells arrests in G1 phase, while most fission yeast cells arrest in G2 phase (Roux et al., 2009). However, the nature of our screen is distinct from screens in budding yeast that have directly assayed CLS, either separately or competitively in mutant pools, and there is limited overlap even among the different budding yeast screens (Breslow et al., 2008; Deutschbauer et al., 2005). It is clear that ageing has a complex genetic basis, and we are far from a unified or complete picture.

Mitochondria and respiration have been implicated in ageing. Cells deleted for the evolutionary conserved 2-oxoglutarate hydrogenase (encoded by *SPBC3H7.03c*), which functions in the Krebs cycle, were long-lived in our assays. Intriguingly, data from worm (Hamilton et al., 2005) point to a direct lifespan benefit through mild impairment of the Krebs cycle. Indeed, impaired respiration slows standard behavioural rates in worm, upregulates genes involved in cell protection, and activates a pathway known as ‘retrograde response’ (Cristina et al., 2009). The reduced function of these enzymes might decrease the tempo or mode of energy generation, which could regulate longevity through mechanisms similar to caloric restriction. On the other hand, cells deleted for the acetyl-CoA transporter gene *SPAC17H9.08* were short-lived. Besides its function in cellular respiration, the acetyl-CoA transporter also functions in the mevalonate pathway, controlled by the Sty1p stress-activated kinase, and is crucial, among others, for membrane maintenance and integrity (Burg et al., 2008). The short lifespan of this transporter mutant might therefore reflect compromised pathways connected to energy status and cell integrity. Cells lacking the succinate dehydrogenase gene *sdh1* were also short-lived. This finding might reflect that Sdh1p is the only enzyme participating in both the Krebs cycle and in the mitochondrial electron transport chain. Thus, similarly to the acetyl-CoA transporter, more than one process is impaired in *sdh1Δ* mutants, which may compromise lifespan. Finally, cells deleted for a short chain dehydrogenase gene (*SPAC521.03*) showed no changes in lifespan. Although, this enzyme is also implicated in respiration, there are 13 short chain dehydrogenases in *S. pombe*, and the absence of phenotype could therefore reflect functional redundancy.

TORC1 is linked with autophagy, which involves vesicle trafficking to recycle cell materials. Loss of two proteins with roles in endocytosis led to long-lived cells: Atg20p, a sorting nexin, and Shd1p, a cytoskeleton-binding protein. A recent report highlights intriguing relationships between neurodegenerative diseases induced by necrosis and endocytosis (Troulinaki and Tavernarakis, 2012).

Proper partitioning of damaged proteins between old and new cells is important for replicative lifespan, both in budding and fission yeast (Erjavec et al., 2008). The partitioning requires the protein aggregation remodeling factor Hsp104p whose overexpression increases replicative lifespan in budding yeast (Erjavec et al., 2007). We found that the *hsp104Δ* mutant had long CLS. This result highlights that the impact of a protein on CLS may be different from that on replicative lifespan.

The relationship between oxidative stress resistance and cellular ageing is much debated in the field. Intriguingly, among the mutant hits identified in our screen, slow growth rather than oxidative stress resistance was associated with longevity. These results suggest that the link between oxidative stress and lifespan (Zuin et al., 2010) might be context-dependent in fission yeast. Our results are consistent with data from worm and flies showing that longevity and stress resistance are not necessarily linked (Blagosklonny, 2008; Doonan et al., 2008), and they are in accordance with the idea that hyper-function of TOR signaling causes ageing (Blagosklonny, 2008; Gems and Doonan, 2009).

## Materials and Methods

### Strains and media

For wild-type control strains, we used 972 *h^−^* or the parental strains for the deletion library, ED666 (*h^+^ ade6-M210 ura4-D18 leu1–32*) and ED668 (*h^+^ ade6-M216 ura4-D18 leu1–32*). The deletion strains for the screen were obtained from the Bioneer version 2.0 library, which includes 3005 mutants (∼84% of all non-essential genes). The *sck2* overexpression strain was from the ORFeome collection (Matsuyama et al., 2006). Cell cultures were grown as indicated in yeast extract plus supplements (YES) unless stated otherwise. Deletion strains were backcrossed to 972 *h^−^* and sequential selections were performed in G418-containing YES agar plates and EMM2 plates (supplemented with lysine for *lys7Δ* strains). Liquid cultures were grown at 32°C with shaking at 130 rotations per minute.

### Drug sensitivity and stress assays

Cells were grown in liquid YES to an OD_600_ of 0.5. Ten-fold serial dilutions of cells were spotted, using replica platers for 48-well or 96-well plates (Sigma), onto YES agar plates, with or without H_2_O_2_ (0.5 mM, 1 mM and 2 mM), KCl (0.5 M and 1 M), caffeine and rapamycin (10 mM and 100 ng/ml, respectively), or Brefeldin A (40 µM and 80 µM). Plates were incubated at 32°C.

### Measurement of cell size at division

Control and drug-treated cells were fixed in 4% formaldehyde for 10 min. at room temperature, washed with 50 mM sodium citrate, 100 mM sodium phosphate, and stained with calcofluor (50 µg/ml). Cells were photographed in a Zeiss microscope using the Volocity acquisition program (PerkinElmer). At least 100 septated cells were counted and analyzed for each condition using the Volocity quantitation package (PerkinElmer).

### High-throughput genetic screening

The haploid deletion library was plated onto YES plates containing G418 using a RoToR HDA robot (Singer). Multiple replicate copies of the library were thus generated. Using the RoToR, the library was compacted into a manageable number of plates and then printed onto plates containing 10 mM caffeine, both singly and in combination with 100 ng/ml rapamycin. Plates containing rapamycin only were not used beyond test experiments, because the mutants showed no differential growth in rapamycin compared to the untreated controls. The plates were incubated at 32°C for 2 days and then manually scored.

### Growth assay

Growth curves under normal and stress conditions were automatically determined by the Biolector microfermentation system (m2p-biolabs), using 48-well flowerplates, at 1.5 ml volume, 1000 rpm and 32°C. The growth dynamics, maximum growth slopes, and maximum cell densities were calculated using the grofit R package (Kahm et al., 2010). In the resulting growth graphs, units of *x*-axis are time (hours) while the *y*-axis shows biomass (arbitrary units) normalized to biomass at time 0.

### Chronological lifespan assay

Cells were grown in YES as described (Roux et al., 2009). When cultures reached a stable maximal density, cells were harvested, serially diluted and plated on YES plates. Colony Forming Units (CFUs) were measured at timepoint 0 at the beginning of the CLS curve (i.e. 100% cell survival). CFU measurements were then conducted daily until cultures reached 0.1–1% of the initial cell survival. Error bars represent standard deviations calculated from three independent repeats, with each sample measured at least three times at each timepoint. The reference strains for CLS assays were ED666 and ED668 with same genetic background as deletion library, which showed similar CLS as wild-type strains. Statistical significance between rapamycin-treated and untreated cultures (Fig. 3; supplementary material Fig. S1) was calculated using one-tailed paired t-tests using data from three biological repeats. Pearson correlations for comparing CLS assays to revival and phloxin B assays (Fig. 4C) were calculated using the R package.

### Western blotting and antibodies

For protein preparations, cells were diluted in 6 mM Na_2_HPO_4_, 4 mM NaH_2_PO_4_.H_2_O, 1% Nonidet P-40, 150 mM NaCl, 2 mM EDTA, 50 mM NaF supplemented with protease (PMSF) and phosphatase inhibitors (Sigma cocktails 1 and 2) together with glass beads. Cells were lysed in a Fastprep-24 machine. Phospho-(Ser/Thr) Akt Substrate (PAS) Antibody (9611, Cell Signaling) for detection of P-S6 (p27) and anti-*rps6* (ab40820, Abcam) were used at 1/2000. Detection was performed using anti-rabbit HRP-conjugated antibody (1/5000 dilutions) with the ECL Western Blotting Detection System (GE Healthcare) according to manufacturer's protocol. Western Blot quantifications have been performed using ImageJ software (http://imagej.nih.gov/ij).

### Quantitative assay for oxidative stress resistance

Verified deletion mutants (hits from screen) were analysed in micro-cultures using the Biolector system according to manufacturer's instructions. Four repeats of the control strain were used. Rapidly growing cultures in YES (OD_600_ = 0.5) were diluted to OD_600_ = 0.15, and 1.5 ml of each culture was then added in each well of a standard flowerplate (m2p-labs). Cells were grown at 1000 rpm at 32°C. All strains were analysed in the presence and absence of 0.5 mM H_2_O_2_ in YES. Readings were taken every 10 min until cultures reached stationary phase. Growth dynamics were analysed using the grofit package from R.

### Polysome profiling

Translational profiles were acquired as previously described (Lackner et al., 2007). Briefly, *S. pombe* protein preparations were performed according to a standard protocol as described above, but using a different lysis buffer (20 mM Tris-HCl pH 7.5, 50 mM KCL, 10 mM MgCl). Linear sucrose gradients (10–50%) were generated using a Biocomp Gradient Master, and protein preparations were centrifuged at 35,000 rpm for 2 h 40 min. Polysome gradients were then loaded to the fractionator to obtain translational profiles. For the calculation of polysome-to-monosome ratios, the AUC of the monosome peak (not including 40S and 60S peaks) and the AUC of polysome peaks were measured with ImageJ package.

### SGA analyses

SGAs were performed as described (Baryshnikova et al., 2010) using the RoToR robot (Singer) settings described for *S. pombe*, with the query mutant *h^−^ sck2::natMX4* and the Bioneer v2.0 haploid library. A control query strain *h^−^ ade6::natMX4* that does not affect the fitness of the mutant library was performed in parallel and used for measurements of strain fitness and colony growth normalization. Scoring was performed both manually and using an in-house developed software pipeline (manuscript in preparation). The fitness of double mutants of the control query SGA (representing the fitness of the library single mutant strains) was calculated as colony sizes. These values were multiplied by the colony-size ratio of *sck2* relative to *ade6* mutations, representing the fitness of the query. The result was divided by the fitness of the double mutants of the *sck2* query. A ratio less than 1 indicated that the double mutant grows worse than the additive combination of the individual single mutants. For our scoring, relative colony-size cutoffs of 0.8 and 1.2 were used for negative and positive interactions, respectively.

### Estimation of enrichment in genetic interactions

To statistically assess the number of documented genetic interactions between the hits, we performed permutation tests as previously described (Zhang et al., 2009). Unique genetic interactions for the genes were downloaded from BioGRID (Chatr-Aryamontri et al., 2013), ignoring interactions found more than once. Ten thousand random sets of 25 genes (same number as genetic interactions annotated in BioGRID among our mutant hits) were checked for annotated genetic interactions. Only 2.7 interactions were found on average, compared to 18 interactions in our set of interest, with only 1 random set showing >18 interactions (p = 0.0001).

## Supplementary Material

Supplementary Material
