# Supplementary material for: Systematic screen for mutants resistant to TORC1 inhibition in fission yeast reveals genes involved in cellular ageing and growth
Source: Biol Open. 2014 Jan 17;3(2):161–71. doi: 10.1242/bio.20147245 (PMC3925319; doi:10.1242/bio.20147245)
Supplement: Supplementary Material [file supp_3_2_161__index.html]

Systematic screen for mutants resistant to TORC1 inhibition in fission yeast reveals genes involved in cellular ageing and growth — Supplementary Material 

# Systematic screen for mutants resistant to TORC1 inhibition in fission yeast reveals genes involved in cellular ageing and growth

## bio.20147245 Supplementary Material

**Files in this Data Supplement:**

- Supplementary Material - Charalampos Rallis et al. doi: 10.1242/bio.20147245
- Table S1 - List of all deletion mutants that are resistant to rapamycin and caffeine treatment in 4 independent repeats of genetic screen.
- Table S2 - List of 33 deletion mutants that are resistant in at least 3 of the 4 independent repeats of the screen, with their CLS phenotypes and *S. cerevisiae* orthologs indicated.
- Table S3 - Lists of all deletion mutants that appear sensitive to caffeine treatment in 4 independent repeats of the genetic screen.
- Table S4 - Summary of CLS presented in Fig. 2.
- Table S5 - Summary of CLS presented in Fig. 3.
- Table S6 - List of positive and negative genetic interactions of sck2Δ, along with Gene Ontology enrichment analysis and statistics.
- Table S7 - Data and statistics for all polysome profiling experiments.
