# Supplementary material for: Systematic screen for mutants resistant to TORC1 inhibition in fission yeast reveals genes involved in cellular ageing and growth
Source: Biol Open. 2014 Jan 17;3(2):161–71. doi: 10.1242/bio.20147245 (PMC3925319; doi:10.1242/bio.20147245)
Supplement: Supplementary Material [file supp_bio.20147245_bio.20147245-s1.pdf]

## Supplementary Material

Charalampos Rallis et al. doi: 10.1242/bio.20147245

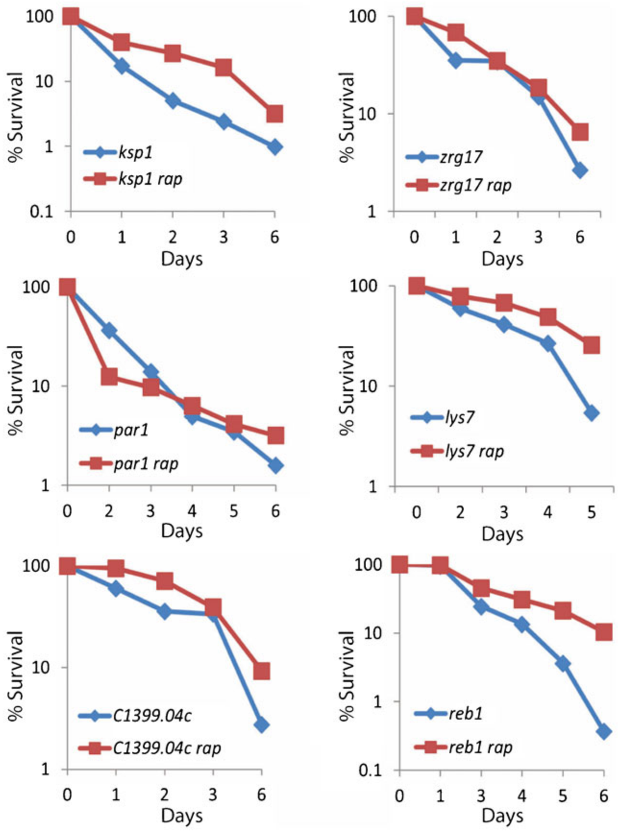


**Fig. S1. Comparison of chronological lifespans of long-lived mutants with (red curves, rap) or without (blue) rapamycin treatment during the growth phase.**

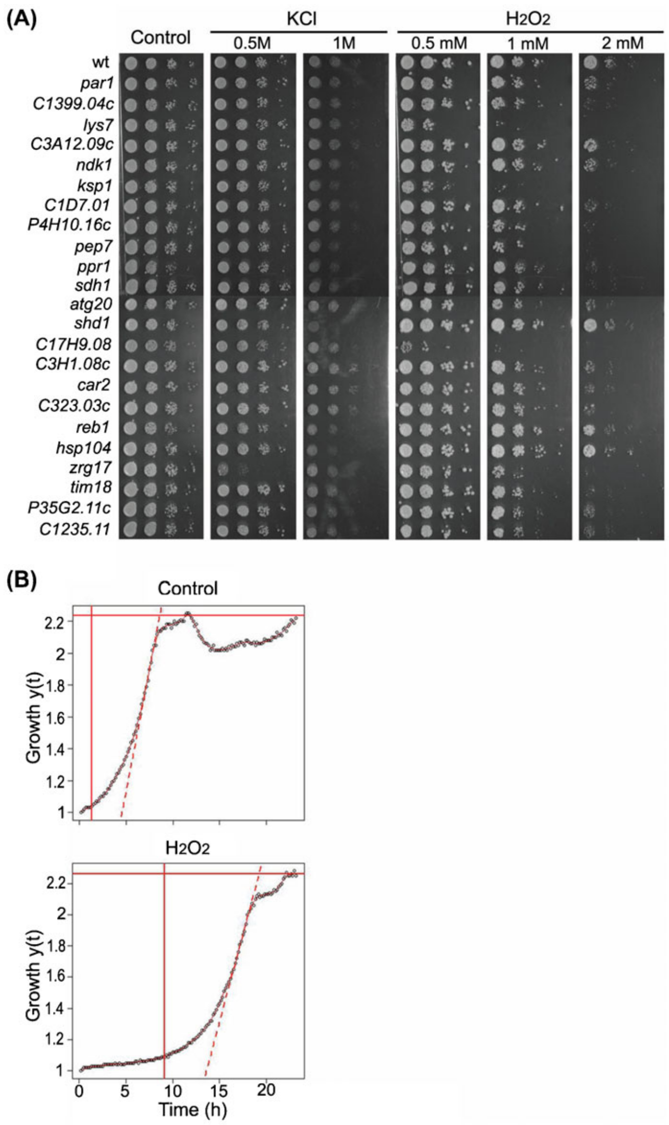


**Fig. S2. Qualitative and quantitative analysis of stress sensitivity phenotypes of mutant cells.** (A) Serial dilutions of mutant cells on plates. In the presence or absence of different concentrations of KCl and $H_2O_2$ as indicated. The wild-type reference strain (wt) and different deletion mutants analysed are indicated at left using common names when available and systematic names otherwise. (B) Analysis of growth kinetics in the absence (top) or presence (bottom) of 0.5 mM $H_2O_2$. Solid red vertical lines indicate the beginning of growth (end of lag phase), and the red hatched lines indicate maximal growth. The *y*-axes are relative to time zero biomass measured by light scattering that measures both number and size of cells.

Biology Open

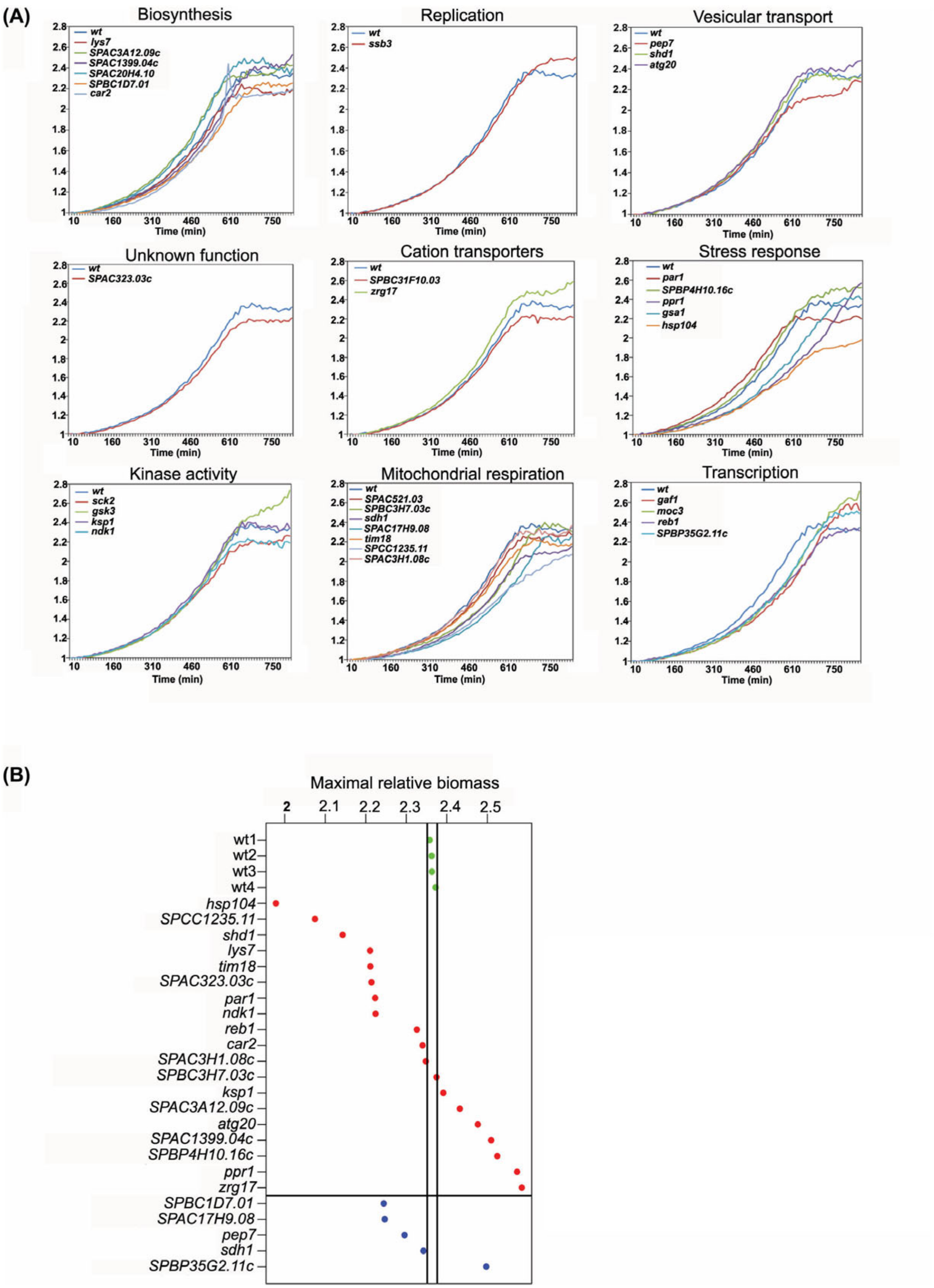


**Fig. S3. Growth and maximum cell density analysis of mutant cells.** Maximum growth densities do not correlate with longevity. (A) Growth patterns of the 33 mutants from our screen. The *y*-axes show arbitrary units relative to time zero biomass that is measured by light scattering and is the result of cell counts and cell size. (B) Long- (red dots) or short-lived (blue dots) mutants can grow to either smaller or larger maximum cell densities than the 4 repeated experiments of wild-type reference cells.
