# Supplementary material for: Systematic screen for mutants resistant to TORC1 inhibition in fission yeast reveals genes involved in cellular ageing and growth
Source: Biol Open. 2014 Jan 17;3(2):161–71. doi: 10.1242/bio.20147245 (PMC3925319; doi:10.1242/bio.20147245)
Supplement: Supplementary Material [file supp_bio.20147245_Table_S1.docx]

**Table S1.**

List of all deletion mutants that appear resistant to rapamycin and/or caffeine treatment in 4 independent repeats of the genetic screen.

**REPEAT 1**

| **Systematic name** | **Common name** | **Description** |
| --- | --- | --- |
| SPCC188.02 | *par1* | protein phosphatase regulatory subunit Par1 |
| SPBC14C8.04 |  | acetolactate synthase regulatory unit |
| SPAC3A12.09c |  | urease accessory protein UreD |
| SPBC19F8.02 |  | nuclear distribution protein NUDC |
| SPAC1687.15 |  | serine/threonine protein kinase Gsk3 |
| SPBP4H10.16c |  | phosphatase activator |
| SPBC16E9.13 |  | serine/threonine protein kinase Ksp1 |
| SPBC1D7.01 |  | prefoldin subunit 1 |
| SPCC1902.01 | *gaf1* | transcription factor Gaf1 |
| SPAC343.18 | *rfp2* | SUMO-targeted ubiquitin-protein ligase subunit Rfp2 |
| SPAP27G11.10c |  | nucleoporin Nup184 |
| SPAC22E12.19 |  | histone deacetylase complex subunit |
| SPAC222.05c |  | COX RNA-associated protein |
| SPAC1952.11c |  | urease |
| SPAC521.03 |  | short chain dehydrogenase |
| SPAC806.07 | *ndk1* | nucleoside diphosphate kinase |
| SPBC31F10.03 |  | ChaC-like protein |
| SPAC1610.02c |  | mitochondrial ribosomal protein subunit L1 |
| SPAC17G6.08 | *pep7* | prevacuole/endosomal FYVE tethering component Pep7 |
| SPAC1399.04c |  | uracil phosphoribosyltransferase |
| SPBP8B7.13 |  | conserved fungal protein |
| SPAC6F6.17 | *rif1* | telomere length regulator protein Rif1 |
| SPAC17C9.02c | *lys7* | alpha-aminoadipate reductase phosphopantetheinyl transferase Lys7 |
| SPBC1539.04 |  | conserved eukaryotic protein |
| SPAC8C9.05 |  | D-Tyr-tRNA deacylase |
| SPAC3G6.05 |  | Mvp17/PMP22 family |
| SPBC17A3.02 |  | conserved fungal protein |

**REPEAT 2**

| SPAC824.07 |  | hydroxyacylglutathione hydrolase |
| --- | --- | --- |
| SPBC1198.11c | *reb1* | RNA polymerase I transcription termination factor Reb1 |
| SPBC83.04 | *apc15* | anaphase-promoting complex subunit Apc15 |
| SPCC188.02 | *par1* | protein phosphatase regulatory subunit Par1 |
| SPAC14C4.11 |  | polyphosphate synthetase |
| SPAC56F8.04c | *coq2* | para-hydroxybenzoate--polyprenyltransferase Coq2 |
| SPBC16E9.13 | *ksp1* | serine/threonine protein kinase Ksp1 |
| SPBC1D7.01 |  | prefoldin subunit 1 |
| SPAC16C9.07 | *ppk5* | serine/threonine protein kinase Ppk5 |
| SPBC947.10 |  | ubiquitin-protein ligase E3 |
| SPCC663.14c |  | membrane transporter |
| SPCC16A11.08 | *atg20* | sorting nexin Atg20 |
| SPAC4G8.03c |  | RNA-binding protein |
| SPBC3H7.03c |  | 2-oxoglutarate dehydrogenase (lipoamide) (e1 component of oxoglutarate dehydrogenase complex) |
| SPBC359.03c |  | amino acid permease, unknown 8 |
| SPCC1902.01 | *gaf1* | transcription factor Gaf1 |
| SPAC821.07c | *moc3* | transcription factor Moc3 |
| SPAC1687.15 | *gsk3* | serine/threonine protein kinase Gsk3 |
| SPBC32F12.02 | *rec14* | recombination protein Rec14 |
| SPBP4H10.16c |  | phosphatase activator |
| SPBC21C3.08c |  | ornithine aminotransferase |
| SPAC17H9.08 |  | mitochondrial coenzyme A transporter |
| SPAC3A12.09c |  | urease accessory protein UreD |
| SPAC20H4.10 | *ufd2* | ubiquitin-protein ligase E4 |
| SPBC16E9.14c | *zrg17* | membrane transporter |
| SPAC3H1.12c | *snt2* | Lid2 complex subunit Snt2 |
| SPBC16D10.08c |  | heat shock protein Hsp104 |
| SPBP35G2.11c |  | transcription related zf-ZZ type zinc finger protein |
| SPAC23G3.12c |  | serine protease |
| SPAC4G8.08 |  | iron ion transporter |
| SPCC4G3.09c | *gyp3* | GTPase activating protein Gyp3 |
| SPAC323.03c |  | sequence orphan |
| SPCC550.03c |  | RNA helicase involved in mRNA catabolism |
| SPAC17A2.09c | *csx1* | RNA-binding protein Csx1 |
| SPAC1556.02c | *sdh1* | succinate dehydrogenase Sdh1 |
| SPBC26H8.03 | *cho2* | phosphatidylethanolamine N-methyltransferase Cho2 |
| SPCPB16A4.05c |  | urease accessory protein UREG |
| SPBPB2B2.19c |  | S. pombe specific 5Tm protein family |
| SPBC337.07c |  | carboxypeptidase |
| SPAC13G6.10c |  | O-glucosyl hydrolase |
| SPAC29B12.10c |  | OPT oligopeptide transporter family |
| SPAC56F8.12 |  | conserved fungal protein |
| SPAPB2C8.01 |  | glycoprotein |
| SPAC4G9.09c | *arg11* | N-acetyl-gamma-glutamyl-phosphate reductase/acetylglutamate kinase |
| SPBC3B8.02 | *php5* | CCAAT-binding factor complex subunit Php5 |
| SPBC16A3.14 |  | mitochondrial ribosomal protein subunit S26 |
| SPCC364.05 | *vps3* | GTPase regulator Vps3 |
| SPBP23A10.16 | *sdh4* | TIM22 inner membrane protein import complex anchor subunit Tim18 |
| SPAC18G6.04c | *shm2* | serine hydroxymethyltransferase Shm2 |
| SPCC297.04c | *set7* | histone lysine methyltransferase Set7 |
| SPAC806.07 | *ndk1* | nucleoside diphosphate kinase |
| SPBC13E7.03c |  | RNA hairpin binding protein |
| SPBP4H10.19c |  | calreticulin/calnexin homolog |
| SPAC21E11.04 | *ppr1* | L-azetidine-2-carboxylic acid acetyltransferase |
| SPAC3F10.04 | *gsa1* | glutathione synthetase large subunit Gsa1 |
| SPACUNK4.15 |  | 2',3'-cyclic-nucleotide 3'-phosphodiesterase |
| SPCC23B6.05c | *ssb3* | DNA replication factor A subunit Ssb3 |
| SPAC16E8.01 |  | cytoskeletal protein binding protein Sla1 family |
| SPAC3H1.08c |  | DUF1640 family protein |
| SPAC1399.04c |  | uracil phosphoribosyltransferase |
| SPAC17G6.08 | *pep7* | prevacuole/endosomal FYVE tethering component Pep7 |
| SPCP20C8.02c |  | S. pombe specific UPF0321 family protein 1 |
| SPBC336.13c |  | mitochondrial inner membrane peptidase complex catalytic subunit 2 |
| SPCC16C4.01 | *sif2* | Sad1 interacting factor 2 |
| SPCC576.14 |  | diphthine synthase |
| SPCC1235.11 |  | conserved eukaryotic protein |

**REPEAT 3**

| SPAC105.03c |  | transcription factor |
| --- | --- | --- |
| SPAC1296.04 | *mug65* | spore wall assembly protein |
| SPBC1198.11c | *reb1* | RNA polymerase I transcription termination factor Reb1 |
| SPCC188.02 | *par1* | protein phosphatase regulatory subunit Par1 |
| SPAC1687.15 | *gsk3* | serine/threonine protein kinase Gsk3 |
| SPAC821.07c | *moc3* | transcription factor Moc3 |
| SPBC21C3.08c |  | ornithine aminotransferase |
| SPAC20H4.10 | *ufd2* | ubiquitin-protein ligase E4 |
| SPAC25G10.03 | *zip1* | transcription factor Zip1 |
| SPBC16E9.14c | *zrg17* | membrane transporter |
| SPAC3A12.09c |  | urease accessory protein UreD |
| SPBC3H7.03c |  | 2-oxoglutarate dehydrogenase (lipoamide) (e1 component of oxoglutarate dehydrogenase complex) |
| SPCC1902.01 | *gaf1* | transcription factor Gaf1 |
| SPBC16D10.08c |  | heat shock protein Hsp104 |
| SPAC323.03c |  | sequence orphan |
| SPBP35G2.11c |  | transcription related zf-ZZ type zinc finger protein |
| SPBC16E9.13 | *ksp1* | serine/threonine protein kinase Ksp1 |
| SPAC1556.02c | *sdh1* | succinate dehydrogenase Sdh1 |
| SPAC26A3.09c | *rga2* | GTPase activating protein Rga2 |
| SPCPB16A4.05c |  | urease accessory protein UREG |
| SPBP23A10.16 | *sdh4* | TIM22 inner membrane protein import complex anchor subunit Tim18 |
| SPAC521.03 |  | short chain dehydrogenase |
| SPCC364.05 | *vps3* | GTPase regulator Vps3 |
| SPBC1685.07c |  | amino acid transporter |
| SPBC31F10.03 |  | ChaC-like protein |
| SPBC2A9.07c |  | zf-PARP-type zinc finger protein |
| SPBC20F10.10 |  | cyclin pho85 family |
| SPAC806.07 | *ndk1* | nucleoside diphosphate kinase |
| SPAC11D3.03c |  | meiotic chromosome segregation protein |
| SPAC21E11.04 | *ppr1* | L-azetidine-2-carboxylic acid acetyltransferase |
| SPAC1399.04c |  | uracil phosphoribosyltransferase |
| SPAC17G6.08 | *pep7* | prevacuole/endosomal FYVE tethering component Pep7 |
| SPAC16E8.01 |  | cytoskeletal protein binding protein Sla1 family |
| SPAC3H1.08c |  | DUF1640 family protein |
| SPAC3F10.04 | *gsa1* | glutathione synthetase large subunit Gsa1 |
| SPCC23B6.05c | *ssb3* | DNA replication factor A subunit Ssb3 |
| SPCC13B11.02c |  | sequence orphan |
| SPAC1B3.07c | *vps28* | ESCRT I complex subunit Vps28 |
| SPCC1235.11 |  | conserved eukaryotic protein |

**REPEAT 4**

| SPAC1556.02c | *sdh1* | succinate dehydrogenase Sdh1 |
| --- | --- | --- |
| SPBC16E9.13 | *ksp1* | serine/threonine protein kinase Ksp1 |
| SPBC1D7.01 |  | prefoldin subunit 1 |
| SPCC1902.01 | *gaf1* | transcription factor Gaf1 |
| SPBC31F10.03 |  | ChaC-like protein |
| SPBC1105.14 | *rsv2* | transcription factor Rsv2 |
| SPAC6G10.06 |  | amino acid oxidase |
| SPAC17H9.08 |  | mitochondrial coenzyme A transporter |
| SPAC3A12.09c |  | urease accessory protein UreD |
| SPBC21C3.08c |  | ornithine aminotransferase |
| SPBP4H10.16c |  | phosphatase activator |
| SPAC20H4.10 | *ufd2* | ubiquitin-protein ligase E4 |
| SPBP8B7.06 | *rpp201* | 60S acidic ribosomal protein P2A subunit |
| SPAC21E11.04 | *ppr1* | L-azetidine-2-carboxylic acid acetyltransferase |
| SPAC1399.04c |  | uracil phosphoribosyltransferase |
| SPAC17G6.08 | *pep7* | prevacuole/endosomal FYVE tethering component Pep7 |
| SPAC3H1.08c |  | DUF1640 family protein |
