# Supplementary material for: Systematic screen for mutants resistant to TORC1 inhibition in fission yeast reveals genes involved in cellular ageing and growth
Source: Biol Open. 2014 Jan 17;3(2):161–71. doi: 10.1242/bio.20147245 (PMC3925319; doi:10.1242/bio.20147245)
Supplement: Supplementary Material [file supp_bio.20147245_Table_S2.docx]

**Table S2.** List of 33 deletion mutants that are resistant in at least 3 of the 4 independent repeats of the screen.

Their CLS phenotypes and S. cerevisiae orthologs are indicated.

| Systematic name | Gene name/description | *S. pombe* CLS | *S. cerevisiae* ortholog | *S. cerevisiae* CLS* | *S. cerevisiae* fitness* |
| --- | --- | --- | --- | --- | --- |
| SPCC188.02 | par1 | Increased | YOR014W | NA | Decreased |
| SPAC1399.04c | uracil phosphoribosyltransferase | Increased | YHR128W | NA | NA |
| SPAC22E12.14c | serine/threonine protein kinase Sck2 | Increased | YHR205W | Mixed outcome | Decreased |
| SPAC1687.15 | serine/threonine protein kinase Gsk3, skp1 | Normal/Increased | YMR139W | NA | Decreased |
| SPAC17C9.02c | alpha-aminoadipate reductase transferase Lys7 | Increased | YGL154C | NA | Decreased |
| SPAC3A12.09c | urease accessory protein UreD | Increased | NA | NA | NA |
| SPAC806.07 | nucleoside diphosphate kinase Ndk1 | Increased | YKL067W | NA | Increased |
| SPBC16E9.13 | serine/threonine protein kinase Ksp1 | Increased | YHR082C | NA | Mixed outcome |
| SPBC1D7.01 | prefoldin subunit 1 | Decreased | YJL179W | NA | Decreased |
| SPBP4H10.16c | phosphatase activator | Increased | YOR043W | Decreased | Decreased |
| SPCC1902.01 | transcription factor Gaf1 | Normal | YFL021W | NA | Increased |
| SPAC17G6.08 | prevacuole/endosomal FYVE tethering component Pep7 | Decreased | YDR323C | NA | Decreased |
| SPAC521.03 | short chain dehydrogenase | Normal | YMR226C | Increased | Increased |
| SPBC3H7.03c | 2-oxoglutarate dehydrogenase (lipoamide) | Increased | YIL125W | NA | Decreased |
| SPAC21E11.04 | L-azetidine-2-carboxylic acid acetyltransferase ppr1 | Increased | YJL127C | Decreased | Decreased |
| SPAC1556.02c | succinate dehydrogenase Sdh1 | Decreased | YKL148C | NA | Normal |
| SPCC16A11.08 | sorting nexin Atg20 | Increased | YDL113C | NA | Increased |
| SPAC16E8.01 | cytoskeletal protein binding protein Sla1 family, Shd1 | Increased | YBL007C | NA | Decreased |
| SPAC17H9.08 | mitochondrial coenzyme A transporter | Decreased | YGR096W | NA | Decreased |
| SPAC20H4.10 | ubiquitin-protein ligase E4 | Normal | YDL190C | NA | Decreased |
| SPAC3H1.08c | DUF1640 family protein | Increased | YFL046W | NA | Decreased |
| SPBC21C3.08c | ornithine transaminase Car2 | Increased | YLR438W | NA | Increased |
| SPBC31F10.03 | ChaC-like protein, predicted cation transport regulator | Normal | YER163C | NA | Increased |
| SPAC323.03c | sequence orphan | Increased | NA | NA | NA |
| SPAC3F10.04 | glutathione synthetase large subunit Gsa1 | Normal | YOL049W | NA | Decreased |
| SPAC821.07c | transcription factor Moc3 | Increased | YDR034C | NA | Decreased |
| SPBC1198.11c | RNA polymerase I transcription termination factor Reb1 | Increased | YDR026C | NA | Decreased |
| SPBC16D10.08c | heat shock protein Hsp104 | Increased | YLL026W | NA | Decreased |
| SPBC16E9.14c | cation diffusion family zinc membrane transporter Zrg17 | Increased | YNR039C | NA | Decreased |
| SPBP23A10.16 | TIM22 inner membrane protein import complex anchor subunit Tim18 | Increased | YDR178W | NA | Decreased |
| SPBP35G2.11c | transcription related zf-ZZ type zinc finger protein | Increased | NA | NA | NA |
| SPCC1235.11 | mitochondrial protein, human BRP44L ortholog | Decreased | YGL080W | NA | Decreased |
| SPCC23B6.05c | DNA replication factor A subunit Ssb3 | Normal | YJL173C | NA | NA |

* Data from SGD
