# Supplementary material for: Systematic screen for mutants resistant to TORC1 inhibition in fission yeast reveals genes involved in cellular ageing and growth
Source: Biol Open. 2014 Jan 17;3(2):161–71. doi: 10.1242/bio.20147245 (PMC3925319; doi:10.1242/bio.20147245)
Supplement: Supplementary Material [file supp_bio.20147245_Table_S3.docx]

**Table S3.** List of all deletion mutants that appear sensitive to caffeine treatment in 4 independent repeats of the genetic screen.

**REPEAT 1**

| SPBC530.01 | gyp1 |
| --- | --- |
| SPAC11G7.02 | pub1 |
| SPBC19G7.10c | topoisomerase II-associated deadenylation-dependent mRNA-decapping factor (predicted) |
| SPBC30B4.03c | adn1 |
| SPAC3G9.08 | png1 |
| SPAC3H8.07c | prefoldin subunit 3 (predicted) |
| SPAC16C9.05 | cph1 |
| SPAC4D7.10c | spt20 |
| SPAC25H1.05 | meu29 |
| SPBC20F10.07 | conserved hypothetical |
| SPBC887.17 | transmembrane transporter (predicted) |
| SPBC2D10.17 | clr1 |
| SPAC14C4.06c | poly(A) binding protein Nab2 (predicted) |
| SPBC1A4.02c | leu1 |
| SPBPB2B2.14c | S. pombe specific DUF999 protein family 8 |
| SPAC1834.10c | sequence orphan |
| SPAC31A2.13c | SNARE Sft1 (predicted) |
| SPAC1610.02c | mitochondrial ribosomal protein subunit L1 |
| SPCC364.03 | 60S ribosomal protein L17 |
| SPBC2D10.12 | Rad23 homolog Rhp23 |
| SPBC56F2.10c | alg5 |
| SPAC9G1.04 | oxa101 |
| SPCC16C4.20c | sequence orphan |
| SPBC725.09c | hob3 |
| SPAC22F8.12c | shf1 |
| SPAC513.03 | mfm2 |

**REPEAT 2**

| SPAC4G9.19 |  | DNAJ domain protein DNAJB family |
| --- | --- | --- |
| SPAC27E2.01 |  | alpha-amylase homolog |
| SPBC16H5.03c | fub2 | SUMO E1-like activator enzyme Fub2 |
| SPAC2F3.12c |  | conserved eukaryotic protein |
| SPCC188.02 | par1 | protein phosphatase regulatory subunit Par1 |
| SPBC3D6.10 | apn2 | AP-endonuclease Apn2 |
| SPBC725.10 |  | tspO homolog |
| SPBC31F10.12 |  | RNA-binding protein Tma20 |
| SPBC2F12.11c | rep2 | transcriptional activator Rep2 |
| SPBC29A10.14 | rec8 | meiotic cohesin complex subunit Rec8 |
| SPBC2F12.12c |  | conserved eukaryotic protein |
| SPAC23E2.01 | fep1 | iron-sensing transcription factor Fep1 |
| SPAC824.04 |  | WD repeat protein |
| SPBC23E6.08 | sat1 | Golgi membrane exchange factor subunit Sat1 |
| SPBC543.10 |  | GET complex subunit |
| SPAC3H5.08c |  | WD repeat protein Wdr44 family |
| SPAC26F1.01 | sec74 | guanyl-nucleotide exchange factor Sec74 |
| SPBC21B10.13c |  | transcription factor |
| SPAC1F8.04c |  | hydrolase |
| SPBC25D12.06 |  | RNA helicase |
| SPAC2C4.17c |  | MS ion channel protein 2 |
| SPAC4F10.02 |  | aspartyl aminopeptidase |
| SPCC613.01 |  | membrane transporter |
| SPAC6F12.09 | rdp1 | RNA-directed RNA polymerase Rdp1 |
| SPAC5H10.11 | gmh1 | alpha-1,2-galactosyltransferase Gmh1 |
| SPAC4F8.10c | stg1 | SM22/transgelin-like actin modulating protein Stg1 |
| SPCC63.02c | aah3 | alpha-amylase homolog Aah3 |
| SPAC4H3.07c |  | protein phosphatase Fmp31 |
| SPCC188.07 | ccq1 | telomere maintenence protein |
| SPCC18B5.01c | bfr1 | brefeldin A efflux transporter Bfr1 |
| SPAC13G7.05 |  | acyl-coA-sterol acyltransferase |
| SPCC74.02c |  | mRNA cleavage and polyadenylation specificity factor complex associated protein |
| SPBP4H10.11c | lcf2 | long-chain-fatty-acid-CoA ligase |
| SPAC30D11.10 | rad22 | DNA repair protein Rad22 |
| SPAC29A4.20 |  | RNA polymerase II elongator complex, histone acetyltransferase subunit |
| SPBC20F10.07 |  | GRAM domain protein |
| SPAC1A6.09c | lag1 | sphingosine N-acyltransferase Lag1 |
| SPAC29B12.05c |  | S-adenosylmethionine-dependent methyltransferase |
| SPAC630.14c | tup12 | transcriptional corepressor Tup12 |
| SPBC14C8.17c |  | SAGA complex subunit Spt8 |
| SPBC428.08c | clr4 | histone H3 methyltransferase Clr4 |
| SPAC959.04c |  | mannosyltransferase complex subunit |
| SPBC3B9.11c | ctf1 | mRNA cleavage and polyadenylation specificity factor complex subunit Ctf1 |
| SPAC1D4.03c | aut12 | autophagy associated protein Aut12 |
| SPAC57A7.07c |  | homocysteine methyltransferase |
| SPBPB2B2.14c |  | S. pombe specific DUF999 protein family 8 |
| SPBC1921.04c |  | sequence orphan |
| SPBC29A10.16c |  | cytochrome b5 |
| SPAC30D11.04c | nup124 | nucleoporin Nup124 |
| SPBC215.03c | csn1 | COP9/signalosome complex subunit Csn1 |
| SPBP35G2.07 | ilv1 | acetolactate synthase catalytic subunit |
| SPBC3H7.09 | mug142 | palmitoyltransferase |
| SPBC56F2.10c | alg5 | dolichyl-phosphate beta-glucosyltransferase Alg5 |
| SPBP18G5.03 | toc1 | sequence orphan |
| SPBC577.11 |  | sequence orphan |
| SPAC144.06 | apl5 | AP-3 adaptor complex subunit Apl5 |
| SPBC1778.05c |  | sequence orphan |
| SPAC1805.07c | dad2 | DASH complex subunit Dad2 |

**REPEAT 3**

| SPBC16A3.07c | nrm1 | negative regulator of MBF |
| --- | --- | --- |
| SPAC30C2.05 | erv14 | cornichon family protein Erv14 |
| SPBC4C3.12 | 01-Sep | fork head transcription factor Sep1 |
| SPBC354.03 | swd3 | WD repeat protein Swd3 |
| SPAC11G7.02 | pub1 | ubiquitin-protein ligase E3 |
| SPBC16A3.08c |  | nuclear telomere cap complex subunit |
| SPBC21B10.10 | rps402 | 40S ribosomal protein S4 |
| SPBC2F12.11c | rep2 | transcriptional activator Rep2 |
| SPAC18G6.15 | mal3 | EB1 family Mal3 |
| SPAC3G9.08 | png1 | ING family homolog Png1 |
| SPBC31F10.09c | nut2 | mediator complex subunit Med10 |
| SPAC3H5.08c |  | WD repeat protein Wdr44 family |
| SPAC16.01 | rho2 | Rho family GTPase Rho2 |
| SPBPJ4664.06 | gpt1 | UDP-glucose-glycoprotein glucosyltransferase Gpt1 |
| SPCC338.16 | pof3 | F-box protein Pof3 |
| SPAC4D7.03 | pop2 | F-box/WD repeat protein Pop2 |
| SPAC5H10.11 | gmh1 | alpha-1,2-galactosyltransferase Gmh1 |
| SPBC146.13c | myo1 | myosin type I |
| SPCC63.02c | aah3 | alpha-amylase homolog Aah3 |
| SPCC1739.14 | npp106 | nucleoporin Npp106 |
| SPAC22A12.07c | ogm1 | protein O-mannosyltransferase Ogm1 |
| SPAC4D7.10c |  | SAGA complex subunit Spt20 |
| SPBP4H10.11c | lcf2 | long-chain-fatty-acid-CoA ligase |
| SPAC1805.14 |  | sequence orphan |
| SPBC17D1.05 |  | sequence orphan |
| SPAC30D11.10 | rad22 | DNA repair protein Rad22 |
| SPCC594.02c |  | conserved fungal protein |
| SPBC20F10.07 |  | GRAM domain protein |
| SPCC757.09c | rnc1 | RNA-binding protein that suppresses calcineurin deletion Rnc1 |
| SPAC20G4.07c | sts1 | C-24(28) sterol reductase Sts1 |
| SPAC1A6.09c | lag1 | sphingosine N-acyltransferase Lag1 |
| SPCC4F11.04c |  | mannosyltransferase complex subunit |
| SPCC794.12c | mae2 | malic enzyme |
| SPAC6F12.06 |  | Rho GDP dissociation inhibitor Rdi1 |
| SPAC630.14c | tup12 | transcriptional corepressor Tup12 |
| SPAC25B8.17 |  | peptidase family A22 |
| SPAC959.04c |  | mannosyltransferase complex subunit |
| SPAC6B12.12 | tom70 | mitochondrial TOM complex subunit Tom70 |
| SPAC977.05c |  | conserved fungal protein |
| SPAC3G9.07c | hos2 | histone deacetylase (class I) Hos2 |
| SPCC1739.05 | set5 | histone lysine methyltransferase Set5 |
| SPAC14C4.06c |  | poly(A) binding protein Nab2 |
| SPBC8D2.18c |  | adenosylhomocysteinase |
| SPAC57A7.07c |  | homocysteine methyltransferase |
| SPAC3C7.14c | obr1 | ubiquitinated histone-like protein Uhp1 |
| SPAC57A10.10c | sla1 | La protein homolog |
| SPBC1921.04c |  | sequence orphan |
| SPBC29A10.16c |  | cytochrome b5 |
| SPAP8A3.07c |  | phospho-2-dehydro-3-deoxyheptonate aldolase |
| SPBC215.03c | csn1 | COP9/signalosome complex subunit Csn1 |
| SPAC31A2.13c | sft1 | SNARE Sft1 |
| SPAC17A5.14 | exo2 | exonuclease II Exo2 |
| SPAC1F12.07 |  | phosphoserine aminotransferase |
| SPBC29B5.01 | atf1 | transcription factor Atf1 |
| SPBC56F2.10c | alg5 | dolichyl-phosphate beta-glucosyltransferase Alg5 |
| SPBC3H7.09 | mug142 | palmitoyltransferase |
| SPAC8F11.02c |  | diphthamide biosynthesis protein Dph3 |
| SPAC17D4.04 |  | tRNA (cytosine-5-)-methyltransferase |

**REPEAT 4**

| SPCC4G3.04c | coq5 | C-methytransferase |
| --- | --- | --- |
| SPAC513.03 | mfm2 | M-factor precursor Mfm2 |
| SPBC1778.05c |  | sequence orphan |
| SPBC1718.03 | ker1 | RNA polymerase I transcription factor subunit Ker1 |
| SPBC2D10.12 | rhp23 | Rad23 homolog Rhp23 |
| SPAP8A3.07c |  | phospho-2-dehydro-3-deoxyheptonate aldolase |
| SPBP4H10.05c | spe2 | S-adenosylmethionine decarboxylase proenzyme Spe2 |
| SPBC1D7.04 | mlo3 | RNA annealing factor Mlo3 |
| SPBC409.16c |  | sequence orphan |
| SPAC14C4.06c |  | poly(A) binding protein Nab2 |
| SPAC5D6.02c | mug165 | sequence orphan |
| SPAC14C4.09 | agn1 | glucan endo-1,3-alpha-glucosidase Agn1 |
| SPAC57A7.08 | pzh1 | serine/threonine protein phosphatase Pzh1 |
| SPCC594.04c |  | steroid oxidoreductase superfamily protein |
| SPBC691.04 |  | mitochondrial ATP-dependent RNA helicase Mss116 |
| SPAC20G4.07c | sts1 | C-24(28) sterol reductase Sts1 |
| SPBP22H7.04 |  | sequence orphan |
| SPCC24B10.11c |  | THO complex subunit 7 |
| SPAC688.11 | end4 | Huntingtin-interacting protein homolog |
| SPAC20G4.08 |  | sequence orphan |
| SPBC3E7.08c | rad13 | DNA repair nuclease Rad13 |
| SPCC1840.05c |  | phosphomannomutase |
| SPBC1778.09 |  | GTPase activating protein |
| SPBC4F6.06 | kin1 | microtubule affinity-regulating kinase Kin1 |
| SPAC922.03 |  | 1-aminocyclopropane-1-carboxylate deaminase |
| SPCC663.01c | ekc1 | protein phosphatase regulatory subunit Ekc1 |
| SPBC2F12.11c | rep2 | transcriptional activator Rep2 |
| SPAC18G6.15 | mal3 | EB1 family Mal3 |
| SPAC3G9.08 | png1 | ING family homolog Png1 |
| SPBC31F10.09c | nut2 | mediator complex subunit Med10 |
| SPBC4C3.12 | 01-Sep | fork head transcription factor Sep1 |
| SPBC354.03 | swd3 | WD repeat protein Swd3 |
| SPAC11G7.02 | pub1 | ubiquitin-protein ligase E3 |
| SPBC16A3.08c |  | nuclear telomere cap complex subunit |
| SPAC1782.11 | met14 | adenylyl-sulfate kinase |
| SPBC26H8.05c |  | serine/threonine protein phosphatase |
| SPBC26H8.12 |  | cytochrome c heme lyase |
| SPAC144.02 |  | transcription factor |
| SPAC1486.04c | alm1 | medial ring protein Alm1 |
| SPBC725.10 |  | tspO homolog |
