# Supplementary material for: Systematic screen for mutants resistant to TORC1 inhibition in fission yeast reveals genes involved in cellular ageing and growth
Source: Biol Open. 2014 Jan 17;3(2):161–71. doi: 10.1242/bio.20147245 (PMC3925319; doi:10.1242/bio.20147245)
Supplement: Supplementary Material [file supp_bio.20147245_Table_S7.docx]

**Table S7**
